# How sweet! Transcription factor CitZAT5 regulates *CitSUS5* and *CitSWEET6* to promote sugar accumulation in citrus fruit

**DOI:** 10.1093/plphys/kiad222

**Published:** 2023-04-17

**Authors:** Yee-Shan Ku

**Affiliations:** Plant Physiology, American Society of Plant Biologists, USA; School of Life Sciences and Centre for Soybean Research of the State Key Laboratory of Agrobiotechnology, The Chinese University of Hong Kong, Hong Kong SAR, China

Sweetness, sourness, and bitterness make up the unique taste of citrus fruit (Ranganna et al. 1983). Sweetness can balance sourness and mask bitterness. Therefore, sweetness is important for regulating the overall fruit taste. The levels of sugars, acids, and flavonoids regulate the fruit sweetness, sourness, and bitterness, respectively (Ranganna et al. 1983). Understanding the regulation of fruit sugar levels is important for designing breeding strategies to obtain citrus varieties having desirable fruit taste.

Sucrose and its component hexoses, fructose and glucose, are major sugars in citrus fruit (Zhou et al. 2018). Sucrose tastes sweeter than glucose but less sweet than fructose (Basso and Serban 2019). Due to the different sweetness, the ratio among sucrose, fructose, and glucose influences the fruit taste. The hexose to sucrose ratio commonly has been used as a criterion for assessing fruit quality (Lado et al. 2018).

In plants, fixed carbon is transported in the form of sucrose from photosynthetic tissues (the source) to nonphotosynthetic tissues such as ripening fruit (the sink). Once it reaches the fruit, sucrose is converted to fructose and glucose by sucrose synthases or invertases (Lowell et al. 1989), and hexose transporters mediate the storage of fructose and glucose (Shiratake and Martinoia 2007). Because fructose is the sweetest, the storage of fructose largely determines the sweetness of fruit. Therefore, genes mediating the hydrolysis of sucrose and the transport of fructose are important regulators of fruit taste.

In this issue of *Plant Physiology*, Fang et al. (2023) showed that the transcription factor (TF) CitZAT5 regulates the expressions of the sucrose synthase gene *CitSUS5* and fructose transporter gene *CitSWEET6* to promote sugar accumulation in citrus fruit (Fang et al. 2023). Arising through spontaneous mutation on a branch of Gongchuan (GC), Youliang (YL) is a bud sport variety with a different fruit sugar profile. Compared with GC fruit, YL fruit has higher fructose and glucose levels, a higher hexose to sucrose ratio, and elevated sucrose synthase cleavage activity. Expression analyses showed that *CitSUS5* and *CitSWEET6* are more highly expressed in YL fruit compared with GC fruit.

Expression analyses and functional characterizations of *CitSUS5* and *CitSWEET6* support their proposed role in regulating fructose and glucose levels, hexose to sucrose ratio, and sucrose synthase cleavage activity. By overexpressing *CitSUS5* in citrus pulp, the authors showed the sucrose synthase cleavage activity of CitSUS5 mediates the elevated levels of fructose and glucose. Using yeast cells and rice protoplasts as models, the authors demonstrated that CitSWEET6 is a fructose transporter located in the plasma membrane. The authors also showed that the overexpression of *CitSWEET6* in fruit pulp promotes increased fructose concentration.

The authors also investigated the TFs that regulate the expression levels of *CitSUS5* and *CitSWEET6*. Based on the transcriptome data, TFs having similar expression patterns with *CitSUS5* and *CitSWEET6* were first identified as candidates. The authors then tested whether these candidates affect the expression of *CitSUS5* and *CitSWEET6*. Using dual-luciferase assays, the authors showed that TFs CitZAT5 and CitNAC47 activated the promoter of *CitSUS5*. Besides *CitSUS5*, CitZAT5 also activated the promoter of *CitSWEET6*. Furthermore, the authors showed that CitZAT5 interacts with CitNAC47 for a synergetic activation effect on the *CitSUS5* promoter (Fig.). Consistent with the higher expression levels of *CitSUS5* and *CitSWEET6* in YL fruit compared with GC fruit, the expression levels of *CitZAT5* and *CitNAC47* were higher in YL fruit than in GC fruit.

**Figure.**
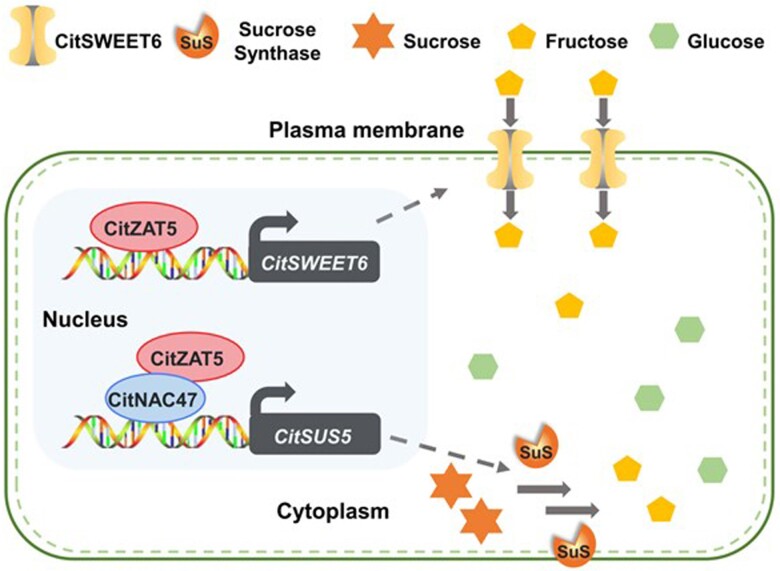
Model of transcriptional activation of *CitSWEET6* and *CitSUS5*. The transcription factors CitZAT5 and CitNAC47 activate the promoters of *CitSWEET6* and *CitSUS5*, respectively. *CitSWEET6* encodes a plasma membrane–localized fructose transporter, and *CitSUS5* encodes a sucrose synthase. *CitSWEET6* expression favors the import of fructose into the cell, whereas expression of *CitSUS5* favors the hydrolysis of sucrose to fructose and glucose inside the cell. CitZAT5 interacts with CitNAC47 to enhance the transcriptional activation effects on *CitSUS5* expression.

The authors also showed that the overexpression of *CitZAT5* on its own was able to upregulate the fruit sucrose, fructose, and glucose contents (Fang et al. 2023). The authors also positively correlated the *CitZAT5* expression level with fruit fructose and glucose contents in different citrus varieties (Fang et al. 2023). ZAT proteins are a subtype of C_2_H_2_-type zinc finger proteins, which have been reported as the regulators of stress tolerance in plants. Interestingly, ZAT proteins have seldom been reported to regulate fruit taste. Although Zhang et al. (2022) reported that AdZAT5 regulates pectin degradation and fruit softening in kiwifruit, they did not report a regulation of sugar accumulation by AdZAT (Zhang et al. 2022). This study by Fang et al. (2023) provided a new insight into the association between ZAT protein and sugar accumulation in fruit (Fang et al. 2023). The authors also provided new insight by showing that ZAT interacts with NAC to regulate sucrose hydrolysis.

To summarize, Fang et al. (2023) showed in this study that the different sweetnesses and hexose to sucrose ratios between YL fruit and GC fruits correspond to different expression levels of *CitSWEET6*, *CitSUS5*, *CitZAT5*, and *CitNAC47* between the two fruit varieties (Fig.). The role of CitZAT5 in regulating the fruit sugar contents suggests possible breeding programs to shape the fruit sugar profiles based on the expression of *CitZAT5*.
